# Participation, challenges and needs in children with down syndrome during cancer treatment at hospital: a qualitative study of parents' experiences

**DOI:** 10.3389/fresc.2023.1099516

**Published:** 2023-04-27

**Authors:** Cathrine Bohnstedt, Margaretha Stenmarker, Linn Olersbacken, Lone Schmidt, Hanne B. Larsen, Kjeld Schmiegelow, Helena Hansson

**Affiliations:** ^1^ Paediatric Oncology Research Laboratory, Copenhagen University Hospital Rigshospitalet, Copenhagen, Denmark; ^2^ Department of Paediatrics and Adolescent Medicine, Copenhagen University Hospital Rigshospitalet, Copenhagen, Denmark; ^3^ Department of Paediatrics, Region Jönköping County, Jönköping, and Department of Biomedical and Clinical Sciences, Linköping University, Linköping, Sweden; ^4^ Institute of Clinical Sciences, Department of Paediatrics, Sahlgrenska Academy at the University of Gothenburg, Gothenburg, Sweden; ^5^ Institute of Clinical Medicine, Faculty of Health Sciences, University of Copenhagen, Copenhagen, Denmark; ^6^ Department of Public Health, University of Copenhagen, Copenhagen, Denmark

**Keywords:** child, adolescent, down syndrome, cancer, participation, assessment

## Abstract

**Background:**

Studies report that it can be challenging to assess and treat side-effects and symptoms among children who have impairments and difficulties in expressing their needs. Children with Down syndrome have an increased vulnerability and an increased risk for contracting leukaemia. There is sparse knowledge about the parental experience of how treatment and side-effects affect children with Down syndrome with leukaemia, as well as the role of participation during treatment.

**Purpose:**

This study aimed to explore the perceptions of parents of children with Down syndrome and leukaemia regarding their child's treatment, side effects and participation during hospital care.

**Methods:**

A qualitative study design was used, and interviews were conducted with a semi-structured interview-guide. Fourteen parents of 10 children with Down syndrome and acute lymphoblastic leukaemia from Sweden and Denmark, 1–18 years of age, participated. All children had completed therapy or had a few months left before the end of treatment. Data was analysed according to qualitative content analysis.

**Results:**

Four sub-themes were identified: (1) Continuously dealing with the child's potential susceptibility; (2) Confidence and worries regarding decisions related to treatment regulation; (3) Challenges in communication, interpretation, and participation; and (4) Facilitating participation by adapting to the child's behavioural and cognitive needs. The sub-themes were bound together in an overarching theme, which expressed the core perception “Being the child's spokesperson to facilitate the child's participation during treatment”. The parents expressed this role as self-evident to facilitate communication regarding the needs of the child, but also regarding how the cytotoxic treatment affected the vulnerable child. Parents conveyed the struggle to ensure the child's right to receive optimal treatment.

**Conclusion:**

The study results highlight parental challenges regarding childhood disabilities and severe health conditions, as well as communication and ethical aspects regarding to act in the best interests of the child. Parents played a vital role in interpreting their child with Down syndrome. Involving parents during treatment enables a more accurate interpretation of symptoms and eases communication and participation. Still, the results raise questions regarding issues related to building trust in healthcare professionals in a context where medical, psychosocial and ethical dilemmas are present.

## 1. Introduction

Children with neurodevelopmental disorders, including Down syndrome (DS), often experience limitations in their autonomy, independence and participation in everyday life due to impairments in adaptive functioning (1, 2). Down syndrome is the most common chromosomal aberration in the Nordic countries and also the most common genetic cause of severe learning difficulties (3). Children with DS are variously affected by for example cognitive impairment (4), delayed language development (5) and more often by behavioural disorders, as compared to the general population (6, 7). When children with neurodevelopmental disorders are diagnosed with severe illnesses, it can be challenging to interpret the child's symptoms and needs (8–10) due to their limited communication ability (11–13).

Children with DS are at risk of developing several medical disorders such as the risk of contracting acute lymphoblastic leukemia (ALL), which in children with DS (DS-ALL) is 20–40 times increased (14, 15) and 1%–3% of all children with ALL also have DS (16). Acute lymphoblastic leukemia is a malignant disease with a significantly improved prognosis in the last decades and today the 5-year survival rate is 80–90 percent (17) Treatment-related side effects are common, such as pain, nausea, fatigue and anxiety (18). In childhood cancer, adequate assessment of treatment's impact on balancing among side-effects and a sufficient degree of treatment is vital, as it can have an impact on survival and quality of life. For children with DS the situation is even more complex. Studies suggest that children with DS-ALL have inferior clinical outcomes (16, 19–22) with higher risk of toxicity, of relapse and of treatment-related mortality (TRM), when compared to children without DS (non–DS-ALL) (19, 23, 24). This vulnerability often leads to reductions in dosage, interruptions of therapy and long hospital stays (24, 25). In a Nordic setting, the research group has previously reported that physicians prescribed significantly lower doses of oral cytotoxic treatment (methotrexate and 6-mercaptopurine) to children with DS-ALL, than they were prescribing to children with non-DS-ALL (25). Presumably, lower doses were prescribed as a way of balancing the treatment related toxicity. This is in line with the recommendations in order to improve the poorer outcome (24). However, these strategies may contribute to an increased risk of relapse and reduced survival rate (25). Still, there is sparse knowledge about how treatment and side-effects affect children with DS-ALL and about the treatment's impact on balancing among side-effects, TRM and a sufficient degree of treatment in the short (24, 25) and long term (26).

To achieve an increased understanding of children with DS living with ALL and severe side effects, it is important to study different aspects of the treatment trajectory, including the parental view of the child's situation. Previous research has shown how parents of children with cognitive impairment become experts on understanding their child's condition and communication behaviour (9, 22, 27). Thus, collaboration with parents is essential during the child's hospitalisations, so that health care professionals can increase their understanding of the child's needs, responses and symptoms (10, 28). Studies are lacking regarding how to map the somatic side effects of DS-ALL in detail and how the child and the parents perceive the problems and the adjustments in treatment. Furthermore, there is a lack of knowledge regarding the influence of hospitalisation on the child's participation in treatment and care (25).

The Nordic results, earlier research regarding the parental role (9, 22, 27) and the fact that it is vital to gain a deeper understanding of the child with DS-ALL and the child's health care needs, inspired the research group to perform the present study. Therefore, the overall aim of this study was to explore the perceptions of parents of children with DS-ALL and regarding their child's treatment, side effects and the child's participation during treatment and hospital care.

## 2. Materials and methods

### 2.1. Design and setting

A national population-based qualitative study design was used in Denmark and Sweden. In Denmark, all children with cancer are treated at four specialized childhood cancer departments. In Sweden, children with malignant disorders are treated at six specialized childhood cancer departments and parts of the treatment, particularly the maintenance therapy, can be administered at local hospitals. At the time of the study, there was a great difference in foetal diagnostics in Denmark and Sweden. In Denmark, all women are and were offered prenatal diagnostic procedures, including detection of a possible DS chromosomal aberration, in the 12th week of and most women accept the offer. A 2,000 people in Denmark have DS. In Sweden, all prenatal diagnostic procedures were voluntary at the time of the study. This approach is still applied in Sweden and different regions within the country have different routines for foetal diagnostics. Approximately 5,000 to 10,000 people in Sweden have DS.

The study was conducted with parents of patients with DS-ALL who were being treated according to the Nordic Society of Paediatric Haematology and Oncology (NOPHO) ALL 2008-protocol. This protocol included four different treatment approaches, i.e., standard risk treatment, intermediate risk treatment, high risk treatment and high risk treatment plus haematopoietic stem cell transplantation. The principals of treatment planning consisted of different parts including induction, consolidation, late intensification and maintenance phases. The treatment could last up to two and a half years after diagnosis. Generally, the same treatment strategies were applied for children with non-DS and DS but with some modifications. These modifications were based on the poor prognosis for children with DS-ALL (4), i.e., children with DS were not eligible for the ALL standard risk treatment, irrespective of white blood cells (WBC) at diagnosis and treatment response. Children with DS-ALL were treated according to the ALL intermediate risk protocol, unless high-risk-criteria were present. The total treatment time was a maximum of two years. The maintenance therapy comprised of oral methotrexate (MTX), oral 6-mercaptopurine (6MP) and intravenous high-dose MTX (HDMTX) with intrathecal injections to reduce the risk of relapse. There were no differences regarding the recommendations for median maintenance therapy blood cell levels, i.e., the therapeutic target for children with DS, in comparison with children with non-DS, were equal. However, maintenance therapy was individually adjusted according to WBC count and side effects.

### 2.2. Sample

Criterion sampling was used to capture a wide insight into parents' perceptions (29). The inclusion criteria were all children with DS-ALL aged 1–18 years, who either had a few months of maintenance therapy remaining or had finished treatment with the NOPHO ALL 2008-protocol within two years. The exclusion criteria were children with relapses or secondary cancers. The population of children with DS-ALL is small in the Nordic countries. During the study period, June 2014 to December 2015, there were 12 children who met the inclusion criteria. All parents were invited to participate, i.e., three families from Denmark and nine from Sweden. Two of the Swedish families were contacted several times with no response. In total, 14 parents of 10 children with DS-ALL participated in the study (Table 1).

**Table 1:** Characteristics of study participants.

Characteristics	*N*
Children with DS-ALL	10
Ethnicity	
Swedish	6
Danish	2
Middle East country	2
Age at time of ALL diagnosis	
1–5	3
5–15	7
Gender	
Female	6
Male	4
Siblings	
Yes	8
No	2
Parents	14
Mothers	8
Fathers	6
Civil status	
Cohabiting	12
Single	2

### 2.3. Data collection

Data was collected using a semi-structured interview-guide that included the following topics: side-effects; treatment adherence and parent/ physician collaboration on dosage regulation; and impact of hospitalisation. The local staff known to each one of the families approached parents in both countries. Interviews were performed in the parents' native languages; in one family, an interpreter was used. The first author (CB) is native Danish-speaking and conducted the interviews in Danish, while the last author (HH), who is native Swedish-speaking and Danish-speaking, conducted the interviews in Swedish. CB and HH used the same interview-guide in respective language and discussed and aligned each other's interviews during the data collection. The parents were interviewed together/alone, with/without their child and siblings, in the family's home (*n* = seven) or by phone (*n* = three) in accordance with the respective parents' preferences. The interviews were audio-recorded with parental permission and transcribed verbatim, including notations of non-verbal expressions. Each of the interviews lasted 30 to 90 min.

### 2.4. Data analysis

The transcribed text was imported to the software program NVivo 10 and analysed using qualitative content analysis according to Graneheim and Lundman (30) comprising four steps in a dynamic process, which moved back and forth between the steps. The process was carried out according to the following principles: (1) reading the transcribed text that constituted the unit of analysis several times in order to obtain an overall understanding of the content; (2) identifying meaning units as words, sentences or paragraphs containing information of interest to the study aim; (3) meaning units were condensed, that is to say, they were shortened while preserving the core, and were labelled with codes. The codes were then compared for similarities and differences and their underlying meaning was interpreted to find commonalities linking them together in sub-themes. In the fourth step, sub-themes were reflected upon and discussed to find the phenomena that connected them to an overarching theme.

To strengthen reliability, the condensed meaning units, sub-themes and themes were discussed and reflected upon by all the authors throughout the analysis process, until the authors reached a consensus. External checks to enhance credibility were also made by considering preliminary interpretations and themes in peer discussions and with collaborating researchers.

### 2.5. Theoretical perspective

The inductive analysis was inspired by the theoretical framework of Participation described by Imms et al. (31) to understand the parental view and the importance of letting the child with DS-ALL have the opportunity to participate and communicate needs and symptoms, verbally and non-verbally. The Concept of Participation is part of a complex multidimensional construct which can be discussed and applied as a process, as well as an outcome (31, 32). Within the family of participation-related constructs (fPRC), participation has been defined with two concepts, i.e., *attendance* in activities and *involvement* while attending the activities (31). The concept of Participation is not applied to the results but to put the parents' perceptions into perspective in the Discussion section.

### 2.6. Ethical considerations

The parents were given written and verbal information about the study's aim, design and procedure and they provided their written consent to participate in the study. Participation was voluntary and all family members were assured confidentiality. None of the authors was involved in the treatment or care of the children. The Danish National Committee on Biomedical Research Ethics (H-4-2014-006) and The Regional Ethics Review Board in Gothenburg, Sweden, (Dnr. 709-14) approved the study.

## 3. Results

Four sub-themes were identified describing the parents' perceptions with their child's treatment, the side-effects and the child's participation during treatment: (1) Continuously dealing with the child's potential susceptibility; (2) Confidence and worries regarding decisions related to treatment regulation; (3) Challenges in communication, interpretation, and participation; and (4) Facilitating participation by adapting to the child's behavioural and cognitive needs. The sub-themes were bound together in an overarching theme, which expressed the parents' core perception of “Being the child's spokesperson to facilitate the child's participation during treatment”. This core perception runs as a common thread of underlying meaning through all the sub-themes showing how parents play a vital role in interpreting and supporting their children with DS who are also undergoing treatment for ALL. The parents regarded themselves as spokespeople for their children by communicating their needs, by sharing information about how the treatment affected the sick child, by standing up for the child's right to receive optimal treatment, and the child's right to attend and be involved in activities whenever possible. The analysis revealed how parents of children with DS-ALL were constantly aware that their child had a vulnerability that required parents to be flexible and solution-oriented to a greater extent than is expected in the corresponding age groups in children with non-DS-ALL. In addition, the parents expressed an ambivalence in the balance between wanting the child to be treated like any other child and the desire for special medical care. The parents' willingness to adapt was reflected in how the parents persistently tried to read their child's verbal and non-verbal forms of expression, to facilitate the child's participation during severe circumstances. The parents' experiences of their child's side effects are presented in Tables 2, 3, and subthemes, codes and supporting quotes are presented in Table 4.

**Table 2:** Parents’ experience of their child's side-effects during induction and consolidation treatment.

Side-effects	Parents (*n*)
Leg pain and loss of walking function	6
Disorders in glucose and electrolytes	4
Lost ability of verbal expression, apathy	4
Nightmares, psychological reaction, hallucinations, depression	3
Allergic reactions	3
Infections	5
Stomatitis	7

**Table 3:** Parents’ experience of their child's side-effects during maintenance therapy.

Patient	Side-effects		
	No	Few	Many
1	x		
2		x	
3		x	
4		x	
5		x	
6			x
7			x
8		x	
9	x		
10		x	

**Table 4:** Illustrations of quotes, codes and sub-themes.

Overarching theme		
Being the child's spokesperson to facilitate the child's participation during treatment		
Quotes	Codes	Sub-themes
“It felt as he [the doctor] was so honest and talked openly with us that they did everything to overcome this, but she is sensitive. And we knew she was sensitive, but also that she is stubborn and fights.” (Mother 4)	Sensitive	Continuously dealing with the child's potential susceptibility
“Because of DS, she will not receive this and this and this. That lead us back to the question about MTX, if one reduces the treatment. It makes me anxious knowing that she gets less treatment than other children.” (Mother 1)	Anxiety insufficient treatment	Confidence and worries regarding decisions related to treatment regulation
“The problem with communication is of course also between the doctors and him, just as well between the nurses and him. That you cannot ask him what he experiences, or it can be difficult to explain to him and make him cooperate when you shall do examinations.” (Mother 7)	Difficulties reading symptoms	Challenges in communication, interpretation, and participation
“When we had been at the ward for a longer time [in the beginning], then there were routines and then she was never sad or anything, when we went back to the ward… Of course, at first when we come in and she has a needle inserted, that is not that nice. She thinks the plaster is the worst part. But after that is done, then it is just, she has taken it so wonderfully and she feels comfortable there. She has been like friends with the nurses. Yes!” (Father 2)	Cooperation during procedures	Facilitating participation by adapting to the child's behavioural and cognitive needs

### 3.1. Continuously dealing with the child's potential susceptibility

Parents had varying experiences of their child's treatment related side effects before maintenance therapy and described a broad range of well-known complications and some of them were severe. An improvement in overall health was the general perception during maintenance therapy and their child's side effects varied from severe to mostly milder or none:

“I cannot tell of any side-effects [during maintenance]. We are at the hospital every eight weeks to receive MTX into his spine, we still did not see anything.” (Mother 8)

“In maintenance phase, she was terribly ill and really low in doses and low in blood values. She was ill a lot. Even though we were on maintenance treatment, we were hospitalised just as often as before. We really just waited for her to get better and stronger, as we were told she should be. As I see it, it just never happened.” (Father 4)

Parents who had experienced their child's severe complications prior to maintenance therapy described experiencing few or no side effects during the maintenance phase as being a relief. However, parents who experienced mild or no side effects prior to and during the maintenance phase raised doubts about the effect of the treatment. For them, side effects could potentially be a sign that treatment was taking effect; thus, no or few complications may be taken as a signal of an insufficient degree of treatment effect.

The parents described that they were eager to adhere to treatment and did not skip oral medication doses because of side-effects or difficulties in the administration. On the contrary, it was essential for them to fulfill treatment with highest possible intensity to keep the child's ALL in remission. The parents described that children who needed daily oral treatment needed to be involved in their own therapy. They tried to make their child to participate, but it was difficult for the child to understand how to swallow and why. The parents did not give up, they struggled to help their child to comply with the treatment as prescribed in order to keep the child's ALL-disease in remission. Still, if the parents failed to help their child to ingest medicine and the child declined to swallow the parents changed strategies to get the child to accept a feeding nasogastric tube. The parents expressed that their perception of their child was more as a fighter than as someone who is frail:

“She struggled through the trajectory, she has been very brave. So, it has worked. But she has been good, because there have been many revolting things she had to swallow. And it has probably not been easy for her, because she started vomiting and so on. But she has struggled through it.” (Father 2)

### 3.2. Confidence and worries regarding decisions related to treatment regulation

Parents expressed that the physicians told them from the outset that children with DS were vulnerable and more susceptible to side effects in comparison with children with non-DS-ALL. They were also informed that the treatment would be regulated according to the therapeutic target, while considering the severity of potential complications and symptoms:

“Our big fear has been if she really gets enough treatment in the end? Will they extend the course? We have discussed those things several times.” (Mother 8)

The parental attitude towards the vulnerability differed, they either wanted their child with DS-ALL to be treated like any other child, or they wanted special medical consideration to be given to their child. Furthermore, this stance could differ between parents within the individual family. The parents valued the doctors' attentiveness and concern, and yet, the parental perceptions were that these treatment regulations were dosage reductions. The parental acceptance and reflections on the consequences for their child's prognosis differed. Some parents did not question regulation but relied on the doctors' expertise, while other parents described how they questioned and lacked a detailed explanation for dosage regulation, i.e., dose reductions:

“We think everything is so great. But the doctors, or some doctors, seems to think… Well, one thinks they are a little too worried and uneasy..…. I do not know how well-informed they are those who are worried. Because we think she is doing so well, being in pre-school and everything and she has not been ill.” (Mother 1)

These actions left the parents with the fear of their child having a relapse due to insufficient treatment, especially when their child did not have or had had any side effects:

“Maintenance treatment is what keeps it suppressed [the leukemia]. So, I remember we were thinking that when she ends treatment, we will have this threat on us at all times that she may relapse.” (Father 4)

Furthermore, their need for explanations were challenged if they felt that the doctor lacked experience and knowledge of children with DS-ALL, leaving the parents with a feeling of insecurity. Some parents even expressed feelings and thoughts regarding that they needed to guard their child's right to participate in the protocol on the same terms as children with non-DS-ALL.

### 3.3. Challenges in communication, interpretation, and participation

The child's ability to communicate varied widely regardless of their age. The child's participation throughout the treatment was built on verbal and non-verbal communication. Some children, who were able to speak, stopped talking during ALL-treatment. This tangible change in the child was a difficult experience for the parents:

“He both used some words and had started with using signs before treatment. But then he did not do any of it. He felt terrible at that time. So, it was immensely frustrating trying to read what he wanted and needed. You cannot ask if he is in pain. He, not now either, says if he is in pain. Even less where it hurts. So, that is a hard part. We understand him a little bit, but the staff has no possibility to ask and get an answer from him.” (Mother 7)

This change also challenged the health care professionals and meant that neither parents nor staff knew how the child experienced side effects such as nausea and pain. The most prominent feature was that the child seldom complained, trying to get the best out of the situation, despite how bad he or she felt. Even when parents saw that the child was suffering from pain, the child would hardly give any expression of doing so. The parents had to interpret their child's symptoms by paying attention to how the child acted, e.g., being uneasy or simply stopping to walk, without saying anything about having pain in the legs. Behavioural changes like these were of great value in the context of the child's limited ability to communicate. Still, the parents depicted the complex task to know the cause of the symptoms, especially because some children lost many of the capabilities they had before starting treatment. This entailed that the parents and the health care professionals sometimes overlooked side effects and symptoms. This meant that the child was not involved in parts of the treatment when the child could have participated and could have had an influence on the handling of the treatment. Although parents considered the interpretation of symptoms and side effects as challenging, they emphasized the importance of utilizing their expertise:

“The doctors did what we said, because we know her best and we know and see how she reacts and feel and what is wrong with her.” (Father 5)

The parents consistently had greater opportunity to interpret and understand their child, despite various limitations, compared to health care professionals.

### 3.4. Facilitating participation by adapting to the child's behavioural and cognitive needs

Changing hospital environments, meeting different health care professionals and diverse procedures adversely affected the child and enhanced the complexity of assessing the child's symptoms. Parents described how their children reacted with behavioural characteristics related to DS during treatment. The degree of such reactions varied, but psychological responses such as apathy and self-injurious behaviour were difficult for the child and the parents to cope with. The parents emphasized that routines and familiar objects and environments with known faces brought comfort and helped their child handle the situation and facilitated cooperation with the health care professionals:

“My son, he is somewhat sensitive. As soon as he hears many sounds, people that come and go, then he gets irritated and angry and hurts himself and son on. He loves music and peace and calm, but we could not really give him that.” (Mother 6)

The child's cognitive impairment also had an influence on the ability to cope with treatment and care. It was difficult for the parents to explain to their child why certain procedures were necessary and one of the mothers described how her child seemed surprised each time she went through well-known procedures. Parents found it crucial to adapt to the child's cognitive level to ease the child's distress and facilitate cooperation by mentally preparing their child before procedures and it was also crucial to let them take the time the child needed. If something went wrong from the start, it was very hard to establish trust again. Information and preparation were therefore vital. Health care professionals who provided trust, information, and preparation and who involved the children were of great value:

“They laugh whilst they give him the medicine, he doesn’t feel anything when he laughs with them. That is why I tell the other doctors to laugh with him while you give him the medicine, then he can handle it better.” (Mother 6)

There was a wide range of responses and the parents emphasized the importance of acknowledging that children with DS have as much need for individual care as children with non-DS. Despite the children's vulnerable situation, a father described how his daughter liked being at hospital, talked to everyone and felt that she was friends with the nurses. The nurses had made great effort to establish the necessary trust with his daughter:

“The way the met her meant a lot, that they were calm, took their time, avoided stress and talked with us. It is quite hard for a stranger to talk with someone with Down syndrome, but just that you really calmly and carefully come closer and talk all the time, helps. I got information in good time before something was going to happen and I could prepare myself and her.” (Father 2)

## 4. Discussion

Children with DS-ALL are reported to be more susceptible to cytotoxic side-effects. This may lead to dose reduction and may affect the cure rate negatively (23, 33–35). On the other hand, assessment of side-effects and symptoms in children with DS can be complex and may potentially induce the risk of not providing the optimal treatment and care to each child. This study provides a deeper understanding of how the cancer treatment affected the children and their possibilities to participate during treatment and hospital care, from their parents' perspective. We found that children with DS-ALL represented a heterogenous group with a varying degree of side-effects, dose reductions and behavioural challenges, needs, and opportunities to be involved during treatment. Parents took on the vital role of being their child's spokesperson to safeguard the child's interest, DS-specific needs and facilitating participation.

Buitenkamp et al. (24) recommend balancing side-effects and dose-reductions, and this study underlines that this balance is a challenging task. Data from our previous study indicate that physicians were less willing to increase the doses in patients with DS-ALL (24); we do not know the reasons for not increasing the cytotoxic treatment, but it might be related to the fear of provoking severe side-effects or from a lack of deep knowledge how to manage and handle DS-ALL. However, the parents described a wide variation in side-effects and treatment interruptions among their children, which questions the common impression about higher susceptibility of suffering side-effects in all children with DS-ALL in comparison with non-DS-ALL. Though, we cannot draw any conclusion based on the interviews with the parents, and an additional study of patient records would be needed. An enhanced attention to the individual varying side-effects and individual needs in dose regulations could improve treatment outcomes for these children (28, 36). In addition, such an approach could stimulate and facilitate each child's opportunities to participation during treatment.

Parents received and perceived the information on susceptibility and dose regulation in different ways. According to the parents in our study, the amount of information offered to them by the physicians on the basis for dose regulations was sometimes limited. This appeared to have a deleterious impact on the parents' understanding of treatment and side-effects and left them feeling insecure and worried about relapses. Furthermore, parents within the same family could have catched and interpreted the information in different ways. According to the Ecoculture theory, from a parental point of view, a family may struggle with their functioning as a uniform system if parents disagree regarding central family matters (37). A family with a child diagnosed with cancer is a family in crisis that needs a uniform system to handle severe circumstances. This fact also serves to underscore the vital importance of parent-physician relationship and communication in paediatric oncology. Moreover, it is important to be aware of the physician-related challenges in this field, i.e., to handle the role of messenger, when it comes to difficult conversations (38). If the knowledge is sparse, like the situation with rare conditions such as DS-ALL, it could be an encounter to inform parents sufficiently throughout the therapy. Furthermore, in an area where there is limited opportunity to gain experience of a condition it can be a big challenge to adapt to fit the needs of the affected child and his/her family (39). In addition, it is also important to consider the context of a study. This study was performed in two Nordic countries in which the organisation within the paediatric community differs and these circumstances could affect the handling of the treatment for the individual child. The results showed that parents wanted the child to have sufficient treatment to avoid relapse, in spite of the fact of potential severe side-effects. To handle this balance, it is important for health care professionals to consider to role of effectively addressing the parents' need for information and explanations. The parents also expressed a need to feel secure that their child with DS had the same opportunities for optimal treatment as children without non-DS. This need is probably grounded in the historical perspective that children with disabilities have been marginalized and their vulnerability can be pronounced (40, 41). According to the *Conventions on the Rights of the Child* (42) and *the Rights of Persons with Disabilities* (43), children with disabilities should be included and involved regarding their care and treatment. Furthermore, parents and health care professionals need to guard each child based on “*the child's best interest”* and each child has “*the right to be heard”* (42) Healthcare professionals need to convey assurance, verbally and non-verbally, that these ethical and medical principals for decisions are taken into account.

In the present study, the results showed the importance of using the parents' expertise in assessing symptoms so that health care professionals better could understand the children's side-effects. Parents described that interpretation of symptoms was challenging, especially because of the child's limited verbal ability. The parents expressed a great responsibility to communicate their children's symptoms to health care professionals. Previous studies have shown that parents of children with limited verbal communication and/or cognitive impairment, including children with DS, often experience that health care professionals have difficulties in recognizing side-effects and symptoms in children with disabilities (11, 12). Parents depict that they are the only ones to identify their child's pain (11, 12). Still, doing so is also a challenging task for them (11, 12). However, the parents did not give up, they struggled to help their child with severe symptoms and to comply with the treatment as prescribed in order to keep the child's ALL-disease in remission. If the parents failed to help their child, e.g., to ingest medicine, and the child declined to swallow the parents changed strategies to get the child to accept a feeding nasogatric tube. According to the literature, parents with a child in need of special care, can develop a strong sense of self-trust and collaboration between parents, as they have to rely on their on way of managing daily impediments (44).

The difficulties encountered in recognizing reactions in the children could be related to the idiosyncratic behaviours of children with cognitive impairments in response to their symptoms and hospitalization and could be related to autistic behaviour that is sometimes associated with DS. The psychological responses as apathy and self-injurious behaviour could be an expression if lacking the possibility to participate (45). Altered behaviour was an important predictor and signal for the parents, when it came to acknowledging the child's symptoms, and these results are similar to findings in other studies (12, 36). Parents felt a great responsibility to pass information and alert the health care professionals regarding the child's changed behaviour. Mental preparation of their child was one way to facilitate cooperation and to decrease stress during procedures. Former studies have illustrated the importance for children with disabilities to have the opportunity for control and choice for participation and being prepared (31). Routines and familiar environments brought comfort and helped the child to handle the situation and facilitated cooperation, which is in line with the Ecocultural theory (37). Thus, meeting the needs of children with DS by utilizing their parents' knowledge about their own child's condition, as well as alterations in their behaviour, may prevent the child from suffering from undetected symptoms (9, 11, 28).

Consequently, it is essential for health care professionals to utilize the parents' expertise, when it comes to arriving at an accurate interpretation of the children's symptoms to provide optimal treatment and care. This could contribute to increasing the sufficiency of the assessment of side-effects, as well, and could serve to prevent overlooking the side-effects, concomitantly enabling the detection of toxicity which may improve the outcome of DS-ALL treatment.

### 4.1. Strengths and limitations

Our study contributes to the sparse knowledge about children with DS-ALL from the parents’ perspectives. Criterion sampling was used due to the small population size of children with DS and ALL on the NOPHO ALL 2008 protocol. All eligible families participated and included children with a broad range of age and communication abilities, as well as a broad range of cognitive and behavioural impairment. The sample was regarded as adequate, since the interviews were information-rich and because saturation could be obtained. Two different countries were included exhibiting organizational and cultural differences, notwithstanding the fact that all of the patients were being treated according to the same protocol. This has increased the diversity and complexity, which may limit the interpretation of the results, but may also enhance the transferability.

Seven interviews were held in parents' homes and three were held as phone interviews. Face to face interviews are conducive to presence and to the interpretation of expressions, but emotions that are difficult to express face to face may better come to light through the somewhat more impersonal phone interview. Our findings should be considered with caution. Nonetheless, they might be transferrable to children with cognitive impairments and limited or to non-verbal children during hospitalisation.

## 5. Conclusion

Our findings suggest that children with DS-ALL present a similar distribution of side-effects and toxicities as compared with children with non-DS-ALL and underscore the importance of physicians' information given to the parents on reduced doses, as this may induce a feeling of greater security about risk of relapse. The children showed a wide variety and variability of reactions and needs related to treatment and hospital admissions.

Parents are in possession of valuable knowledge about their children's health conditions, and involving parents enables a more accurate interpretation of symptoms. Considering the needs of a familiar environment, routines and mental preparation among children with DS-ALL may furthermore decrease their burden. These findings suggest individualised treatment and care to improve the outcome and prognosis for children with DS-ALL and to better accommodate their needs.

## Data Availability

The raw data supporting the conclusions of this article will be made available by the authors, without undue reservation.
